# Mechanism of Pit Formation on Surface of Superconducting Niobium Cavities During Buffered Chemical Polishing

**DOI:** 10.3390/ma18040865

**Published:** 2025-02-16

**Authors:** Zheng Wang, Jinfang Chen, Yawei Huang, Yue Zong, Shuai Xing, Jiani Wu, Xiaowei Wu, Pengcheng Dong, Runzhi Xia, Xiaohu Wang, Xuhao He, Miyimin Zhao, Zhaoxi Chen, Xuerong Liu, Dong Wang

**Affiliations:** 1 Shanghai Institute of Applied Physics, Chinese Academy of Sciences, Shanghai 201800, China; wangzheng@sinap.ac.cn; 2 University of Chinese Academy of Sciences, Beijing 101408, China; 3 Shanghai Advanced Research Institute, Chinese Academy of Sciences, Shanghai 201204, China; zongy@sari.ac.cn (Y.Z.); xings@sari.ac.cn (S.X.); wujn@sari.ac.cn (J.W.); dongpc@sari.ac.cn (P.D.); wangd@sari.ac.cn (D.W.); 4 Center for Transformative Science, ShanghaiTech University, Shanghai 201210, China; huangyw2@shanghaitech.edu.cn (Y.H.); xiarzh@shanghaitech.edu.cn (R.X.); wangxh4@shanghaitech.edu.cn (X.W.); hexh2022@shanghaitech.edu.cn (X.H.); zhaomym2022@shanghaitech.edu.cn (M.Z.); chenzhx@shanghaitech.edu.cn (Z.C.); liuxr@shanghaitech.edu.cn (X.L.); 5 Zhangjiang Laboratory, Shanghai 201210, China; wuxw@zjlab.ac.cn

**Keywords:** superconducting cavity, buffered chemical polishing (BCP), pit, bubble, niobium sample, mechanism

## Abstract

Superconducting radio-frequency niobium cavities processed using buffered chemical polishing (BCP) sometimes show typical W-shaped pits on their surface, which may greatly limit their performance. However, the causes of such pits and effective solutions are not fully understood. In this study, we reproduced the formation of W-shaped pits on the cavity surface through niobium sample BCP experiments, directly observed the sample surface’s evolution during the polishing process and the polished surface’s morphology, and analyzed the cause of W-shaped pits in detail: the formation and attachment of bubbles on the niobium surface during the BCP process. Then, we systematically investigated the effects of different process parameters on the bubbles and pits, including the acid ratio, temperature, and flow rate. We also investigated how the formation of bubbles and pits was affected by the Nb facing orientation and grain size. This study provides insights into the mechanisms by which bubbles and W-shaped pits are formed on niobium surfaces, and highlights possible directions for reducing pit defects in Nb cavities processed using BCP treatment.

## 1. Introduction

Superconducting radio-frequency (SRF) cavities are the main particle-accelerating structures in many modern accelerators, which are widely applied in light sources, particle colliders, spallation neutron sources, etc., due to their ultra-low surface resistance ($R_{s}$) and high acceleration gradient ($E_{acc}$) [1,2,3]. Currently, the most widely used superconducting cavities are generally made of high-purity niobium with RRR ≥ 300. Cavity manufacturing processes may result in damage layers, contamination, and defects on the cavities’ inner surfaces, which may severely limit RF performance [1,2,4,5]. During SRF cavity treatment, surface damage layers, contaminants, and defects are typically removed through chemical polishing, which mainly consists of electropolishing (EP) and buffered chemical polishing (BCP) [1,2,6,7].

BCP is a surface-processing technique that reacts a certain volume ratio of hydro-fluoric acid (HF), nitric acid (${\mathrm{HNO}}_{3}$), and phosphoric acid ($H_{3}{\mathrm{PO}}_{4}$) mixture with niobium to form soluble niobium compounds to achieve the polishing of superconducting cavity surfaces. The nitric acid oxidizes the surface of metallic niobium and produces a niobium oxide film; then, the hydrofluoric acid reacts with the niobium oxide layer to generate soluble oxofluoride acidic species, while the phosphoric acid is mainly used to reduce or control the reaction rate [8,9,10,11]. The commonly used acid volume ratio for superconducting niobium cavity BCP is HF (49%)/HNO_3_ (69%)/H_3_PO_4_ (85%) = 1:1:2, and an acid temperature < 15 °C, acid flow velocity ∼1 cm/s, and etching rate ∼1–3 μm/min are typically used [7,9,11,12]. BCP is widely used in the treatment of various cavities, especially those with asymmetric or complex geometries, due to its simple setup and fast process [13,14].

Well-controlled BCP can achieve rather good smoothness on the surface of superconducting niobium cavities, but sometimes, a peculiar type of pit defect can be found on some cavities, distributed all over the inner surfaces [15,16]. The morphology of these pits is different from that of typical etch pits caused by crystal defects and impurities [17,18]. These defects resemble a wide pit with a bump superposed near the center when viewed under an optical inspection system for superconducting cavities [15]. This phenomenon has been occasionally observed in other labs, such as JLAB, FNAL, DESY, INFN, KEK, and IHEP [19,20,21,22,23,24,25]. Such pit defects may lead to the early quenching of the cavity and greatly limit its performance [19,21,23], resulting in a lower maximum acceleration gradient [1,26,27,28,29] and lower $Q_{0}$ at higher fields [30,31,32]. The field enhancement factor of such pits may reach as high as four in the worst case [33,34].

Some possible sources of pit formation during BCP have been reported in previous studies, such as defects [17], material impurities [18], inclusions [35], and bubbles [36]. However, the exact causes of such pits and their effective solutions are not fully clear, especially the influence of the acid ratio.

This study investigated the causes of pit formation on the surface of superconducting cavities and niobium samples during the BCP process, and the effects of process parameters on the pits, through direct observation of the BCP processes and the optical inspection and measurement of the polished sample surfaces. Based on these experiments, we propose a formation mechanism for bubbles and pits, as well as two efficient methods to mitigate pitting.

## 2. Experiments and Results

### 2.1. Pits on the Cavity Surface

**Figure 1:**
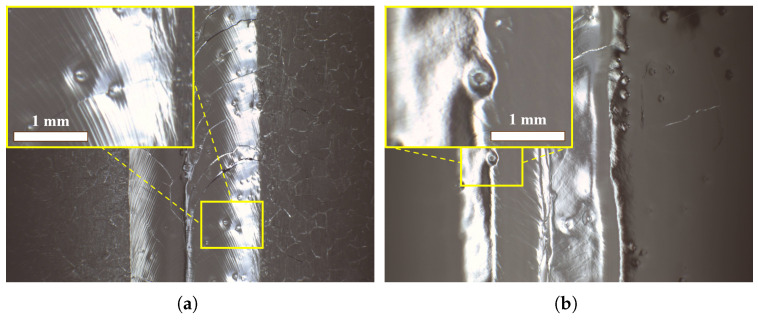
Inner surfaces of 1.3 GHz 9-cell fine-grain cavity HJ006 after BCP-I 60 μm (**a**) and 1.3 GHz single-cell large-grain cavity L03 after EP 175 μm + BCP-I 115 μm (**b**), showing number of peculiar pits.

BCP can be used to etch the inner surface or outer surface of an SRF cavity. The peculiar defects were first noticed on the outer surface of a 1.3 GHz single-cell fine-grain niobium cavity after outer-surface light BCP (BCP-O) in our lab, which was employed to improve the heat transfer performance (Kapitza conductance) of the niobium at the interface with liquid helium (Nb/LHe) [1,37], and to reduce the possible contamination of the cavity inner surface and furnace during cavity heat-treatment. Figure 1 shows the pit defects on the inner surfaces of two cavities after inner-surface BCP (BCP-I).

**Figure 2:**
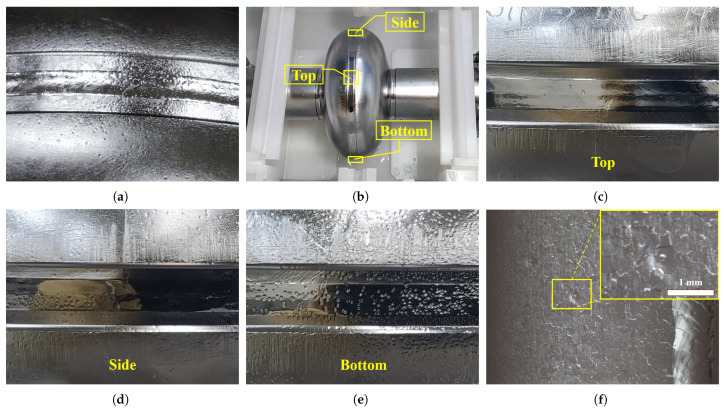
(**a**) Outer surface of 1.3 GHz single-cell fine-grain cavity SS001 after BCP-O 10 μm. (**b**) Placement status of 1.3 GHz single-cell large-grain cavity L04 in BCP-O polishing bath and (**c**–**e**) pits in different regions. (**f**) Inner surface of 9-cell fine-grain cavity HJ006 after BCP-I 60 μm.

We performed BCP experiments at the SHINE surface-treatment platform in Wu-xi (hereafter abbreviated to the Wuxi Platform) [38]. The main polishing process parameters used for a fine-grain cavity (SS001) BCP-O are as follows: an acid volume ratio of HF (49%)/${\mathrm{HNO}}_{3}$ (69%)/$H_{3}{\mathrm{PO}}_{4}$ (85%) of 1:1:2, acid temperature of 8 °C, polishing flow rate of 5 L/min, and polishing time of 6 min. However, the outer surface of the SS001 cavity after polishing showed dense pits with a diameter less than 1 mm, as shown in Figure 2a. The typical shape of the pit was a wide pit with a U-shaped pit superposed near the center. To verify that these pits were produced during the BCP-O process, we carried out BCP-O with another single-cell large-grain cavity L04 twice using the same process, which reproduced the pit defect results.

We examined the surfaces of cavities that had previously undergone BCP-O or BCP-I and found that the distribution of pits on the outer surface of the SRF cavities after BCP-O was as follows: (1) a large number of pits were mainly located in the equator and nearby regions, as well as in the beam tubes, whereas the cavity wall region between the equator and iris was relatively sparse; (2) the distribution of the pits was related to the orientation of the cavity surfaces when the cavity was placed in the BCP-O bath. As shown in Figure 2, the pits on the bottom side of the cavity were the densest; those on the top were the rarest and sporadic, while those on the two sides were in between. For the BCP-I cavities, pits were also found in almost all regions of the inner surface; however, the wall regions of the fine-grain cavities were relatively inconspicuous due to surface roughness (see Figure 2f).

In order to determine the cause and solution for pits formed on the surface of niobium cavities after BCP, we performed BCP experiments with niobium cavities, caps, foils, and samples. These experiments showed that decreasing the acid temperature could slightly reduce the pitting problem, but increasing the acid flow rate within a certain range had no significant effect. We found that BCP experiments using a Nb sample were most appropriate, not only mimicking the SRF cavity BCP, but also allowing us to directly observe the BCP process on the Nb surface. Considering the low cost of sample experiments and the easier characterization of the sample surface, the sample BCP setup is therefore an excellent tool for studying the problem of pits.

### 2.2. Sample Preparation and Sample BCP Setup

Nb samples with dimensions of 8 mm × 5 mm × 3 mm were cut using a wire cutting machine from a Ningxia high-purity (RRR ≥ 300) large-grain niobium sheet or fine-grain niobium sheet (grain size ASTM 5.0∼6.0, namely 64∼45 μm) used for cavity fabrication. After cutting, the small samples were ultrasonically cleaned to remove surface contaminants and dried in an ISO7-grade clean room. The samples were then number-marked on the side using an electro-engraving pen as NLxx (smooth-surface Ningxia large-grain sample, Ra = 0.535 μm), Fxx (smooth-surface Ningxia fine-grain sample, Ra = 0.191 μm), and NFxx (slightly roughened-surface Ningxia fine-grain sample, Ra = 0.925 μm). The slight roughness differences between the three types of samples were from the original niobium sheets. Most of the NLxx samples were single-crystal samples. Before BCP, the samples were weighed using an electronic balance, their thicknesses were measured using a micrometer, and they were photographed using a high-definition camera in the clean room to record the state before polishing.

**Figure 3:**
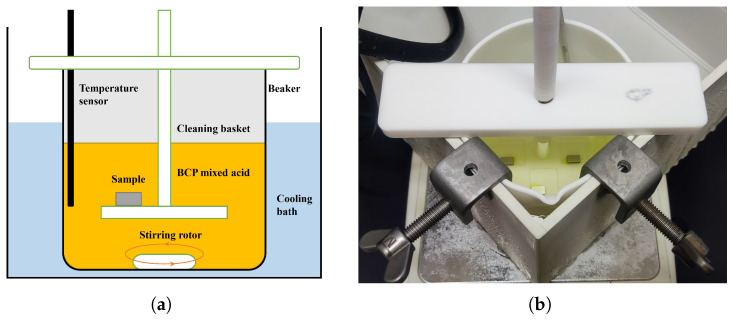
(**a**) Schematic diagram and (**b**) photo of sample BCP setup at Wuxi Platform.

A self-developed sample BCP setup at the Wuxi Platform is shown in Figure 3. This setup is mainly used for sample preparation and sample BCP experiments; it has the basic functions of an SRF cavity BCP facility, such as the ability to control the acid temperature, flow velocity, etc., and can polish multiple samples simultaneously. The setup mainly includes polishing, water cooling and temperature control, acid temperature monitoring, acid flow velocity control, sample cleaning, and other sections. The polishing section consists of a beaker and a cleaning basket, on which the sample is placed to react with BCP acid in the beaker to achieve chemical polishing. The water cooling and temperature control section consists of a cooling water bath, a refrigeration system, and fixed clamps to allow water cooling and acid temperature control of the acid in the beaker. The acid temperature-monitoring section consists of an acid-resistant thermometer and a controller to monitor the temperature of the acid solution in the beaker in real time. The acid flow velocity control section consists of a magnetic stirrer and rotor, which can be set to different rotational speeds, and different rotors can be selected to change the flow velocity of the acid in the beaker. The entire sample BCP setup is placed in a fume hood, which allows the acid gas evolved during polishing to be quickly pumped away and sent to the waste gas treatment system.

### 2.3. Formation of Surface Bubbles and Pits

In order to investigate the mechanism by which the pits on the niobium surface were formed during the BCP process, we selected a group of niobium samples, which consisted of a piece of smooth-surface large-grain NL131, a piece of smooth-surface fine-grain F131, and a piece of rough-surface fine-grain NF131. These samples were placed together in the sample BCP setup to be polished for 5 min, and then were turned up and down to be polished for 5 min (noted as 5 min × 2), where we aimed to ensure regular and symmetrical polishing. The evolution of the sample surfaces was recorded with a camera mounted on a holder throughout the process.

To mimic the actual surface conditions of cavity BCP-I as much as possible, the sample BCP parameters were set as follows: an acid volume of 400 mL in the beaker, acid ratio of HF (49%)/${\mathrm{HNO}}_{3}$ (69%)/$H_{3}{\mathrm{PO}}_{4}$ (85%) of 1:1:2 by volume, acid temperature of 8 °C, rotor rotation speed of 300 RPM (corresponding to an acid flow velocity on the sample surface of around 1.3 cm/s), and polishing time of 5 min × 2. After BCP, the samples were rinsed with ultra-pure water until neutral. The samples were then rinsed again with ultra-pure water in an ISO7 clean room and blown dry with nitrogen.

**Figure 4:**
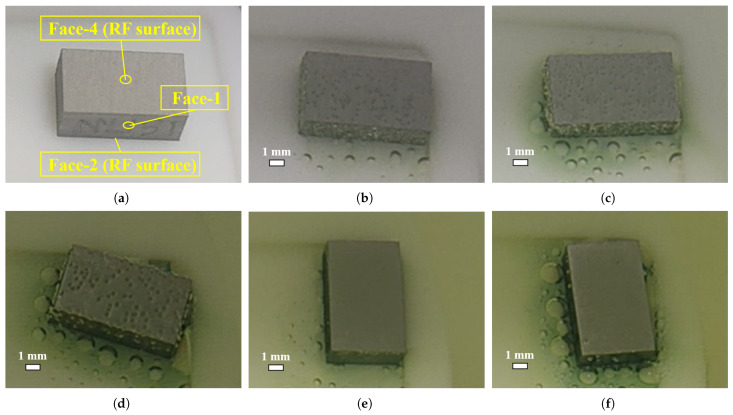
The first 5 min of the BCP process for the large-grain sample NL131, in which many bubbles formed and attached on the top surface (face-4). (**a**) Before immersion in acid; (**b**) after polishing in acid for 30 s, (**c**) 90 s, and (**d**) 270 s. The second 5 min of the BCP process for the large-grain sample NL131, in which only 2 bubbles attached on the top surface (face-2) after polishing for (**e**) 30 s and (**f**) 240 s.

Many bubbles formed and attached to the surfaces of all three samples during the sample BCP; the surface bubble evolution for the large-grain sample NL131 is shown in Figure 4. For ease of description, the face-2 and face-4 of the samples (i.e., RF surfaces) are defined as shown in Figure 4a. During the first 5 min of BCP, when sample NL131 started to react with the acid, dense small dot-like bubbles rapidly appeared on its surface, and these bubbles were approximately uniformly distributed on the surface, as shown in Figure 4b. As the reaction continued, the volume of these bubbles gradually increased, while some bubbles detached from the surface and entered the acid solution; therefore, the number of bubbles on the niobium surface also gradually decreased, but the volume of individual bubbles continued to increase, as shown in Figure 4c. When the bubbles increased to a certain size, they detached from the sample surface and left an imprint at the detachment location, as shown in Figure 4d. Furthermore, we noticed that few new bubbles were formed in the subsequent stages except for the initial stage of bubble formation. It is very interesting that, within the second 5 min of BCP after flipping the sample up and down, there were likewise very few bubbles formed on the samples’ surfaces, as shown in Figure 4e,f. The reason may be the removal of the highly reactive superficial layer of the initial few microns on the surface, which has a large etching rate [8,9,39].

We also observed two strange phenomena during the sample BCP process (see Figure 4): (1) As the polishing proceeded, a green product layer was gradually formed on the sample’s surface. When this product was dissolved, the acid solution in the beaker also gradually turned to a yellow-green or yellow color. It has been shown that the products were different fluorinated species [9], such as ${\mathrm{NbF}}_{4}$ [40]. (2) There was a transparent annular region around the bubbles with no green liquid layer.

**Figure 5:**
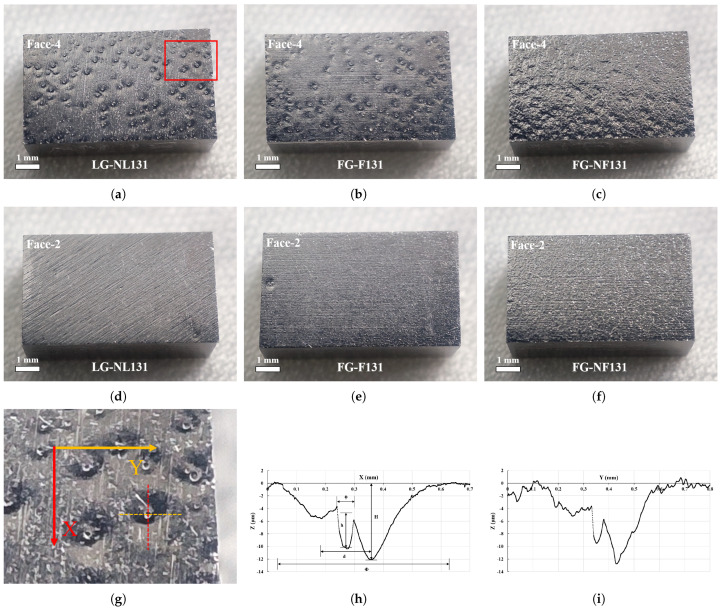
(**a**–**f**) Surface morphology of face-4 (**up**) and face-2 (**down**) for the large-grain sample NL131, fine-grain sample F131, and slightly rough fine-grain sample NF131 after BCP for 5 min × 2. (**g**) Magnified view, and (**h**) X and (**i**) Y direction profiles of the pit marked by the red box on face-4 of the large-grain sample NL131, as measured using a stylus profiler (Alpha-step D-600, made by KLA, Milpitas, CA, USA).

Next, surface optical inspection of the polished samples was carried out in the clean room using a high-definition camera, and the surface morphology of the large-grain sample NL131, fine-grain sample F131, and slightly rough fine-grain sample NF131 is shown in Figure 5. Pits such as the cavity surface (see Figure 1) were clearly formed on the surfaces of all three samples after BCP for 5 min × 2. The shape of the pits on face-4 (corresponding to the top surface during the first 5 min of BCP) of the large-grain sample NL131 and fine-grain sample F131 is clear and regular, while the shape of the pits on face-4 of the slightly rough fine-grain sample NF131 is vague and irregular.

The morphology of these pits is characterized by a spherical or conical bump in the middle of a large circular pit, and a very small point pit on top of the bump, as shown in Figure 5g. The cross-sectional morphology of the pit (marked with a red box) on the surface of the large-grain sample NL131 measured using a stylus profiler is shown in Figure 5h,i; the pit cross-sectional profile overall shows a W-shape, with a small U-shape in the middle vertex of the W. The diameter of the large circular pit is about 0.61 mm, the maximum depth of the pit is about 12.1 μm, and the bottom diameter of the middle bump is about 0.17 mm, while the diameter and depth of the small point pit are about 0.06 mm and 5.5 μm, respectively, as seen in the x-direction profile in Figure 5h. Surprisingly, the depth of the pits is much smaller than the transverse dimension, even though they look very deep from the high-resolution photographs.

A comparison of the pits on the three samples is provided in Table 1. We can see that the number, size, depth, and shape of the pits on the surfaces of the three samples were nearly the same. In addition, face-2 of the sample (corresponding to the bottom surface for the first 5 min of BCP, and the top surface for the second 5 min of BCP after flipping the sample) also had similar pits, but the number of them was very low, as shown in Figure 5d–f.

**Figure 6:**
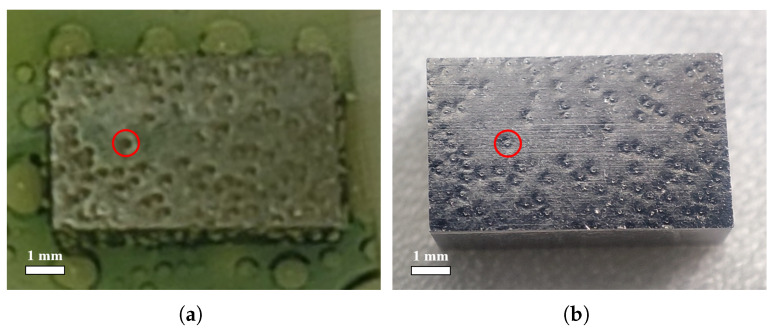
(**a**) The position and size of the bubbles during polishing (t = 270 s) and (**b**) the W-shaped pits after polishing on face-4 of the fine-grain sample F131. A bubble (**left**) and its corresponding W-shaped pit (**right**) are marked in red circles, respectively.

The bubble formation on the sample surface during the BCP process and the presence of W-shaped pits after polishing naturally suggests a direct correlation. Figure 6 compares the position and size of the bubbles on the surface of the above fine-grain sample NF131 and the pits on the surface after polishing. It is obvious that the position and size of the bubble and the transparent annular region around the bubble correspond to the position and size of the middle bump and the large pit of the pit. Therefore, the direct cause of the W-shaped pits on the sample or superconducting cavity surface after BCP is the formation and attachment of bubbles on the niobium surface during the BCP process.

### 2.4. Effect of BCP Process Parameters on Pits

The main controllable process parameters for cavity BCP are the mixed acid ratio, acid temperature, and acid flow rate, in addition to the different effects of the niobium cavity grain size and polishing surface orientation. It is important to study the effects of these parameters on bubbles and pits, with the purpose of suppressing or even avoiding the formation of pits by adjusting the process parameters, to obtain a better polished surface. Moreover, this can also drive us to further optimize the BCP process for superconducting cavities, aiming to improve their superconducting RF performance.

#### 2.4.1. Phosphoric Acid Ratio

Phosphoric acid ($H_{3}{\mathrm{PO}}_{4}$) is used to adjust the polishing rate of the BCP process. To investigate the effects of the phosphoric acid ratio on bubbles and W-shaped pits, we fixed the HF/${\mathrm{HNO}}_{3}$ ratio at 1:1 and sequentially changed the phosphoric acid ratio to 1, 2, 2.4, 4, 6, and 8, respectively. Then, the other experimental parameters were kept unchanged as before: an acid temperature of 8 °C, rotor speed of 300 RPM, polishing time of 5 min × 2, etc. Three samples for each group of experiments (including one each of slightly rough fine-grain NF, large-grain NL, and fine-grain F) were simultaneously polished on the cleaning basket, and the sample polishing process was observed and recorded with a camera. The same experimental conditions were used in the subsequent experiments unless otherwise specified.

**Figure 7:**
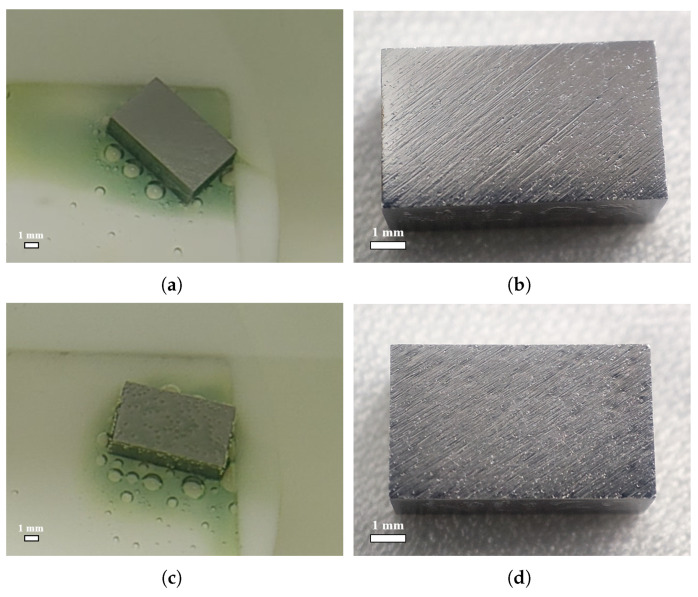
Acid ratio of 1:1:1: (**a**) surface state of face-4 after polishing for 90 s in first 5 min of BCP; (**b**) surface morphology of face-4 after polishing for large-grain sample NL123. Acid ratio of 1:1:2.4: (**c**) surface state of face-4 after polishing for 270 s in first 5 min of BCP; (**d**) surface morphology of face-4 after polishing for large-grain sample NL133.

For a visual comparison with the acid ratio of 1:1:2, Figure 7 shows the samples’ surfaces after polishing with ratios of 1:1:1 and 1:1:2.4. A ratio of 1:1:1 is another commonly used BCP mixed acid ratio in addition to 1:1:2. As one can see in Figure 7, with a ratio of 1:1:1, no bubbles or W-shaped pits formed on the sample surface. It should be emphasized that the pits studied in this work were those caused by bubble attachment, not small pitting pits caused by crystal defects or impurities [17,18]. With a ratio of 1:1:2.4, a large number of bubbles were formed and adhered to the sample surface, but the pits on the polished surface were not obviously prominent; most of the area had only shallow pit imprints, and some of them were even unrecognizable. This further confirms that niobium surface bubble attachment leads to the formation of W-shaped pits.

The pit depth is related to the etching rate and etching time. The single-sided polishing rate of the large-grain sample NL133 calculated from the polishing removal mass was about 1.05 μm/min (corresponding to 1:1:2.4), while the single-sided polishing rate of the large-grain sample NL131 was about 1.21 μm/min (corresponding to 1:1:2). For the same bubble attachment time of 5 min, the maximum depth of the NL133 pits was about 7 μm, while the maximum depth of the NL131 pits was about 12.1 μm. It is obvious that an increase in phosphoric acid ratio reduced the etching rate. Table 2 presents the polishing rates of large-grain samples at different phosphoric acid ratios.

**Figure 8:**
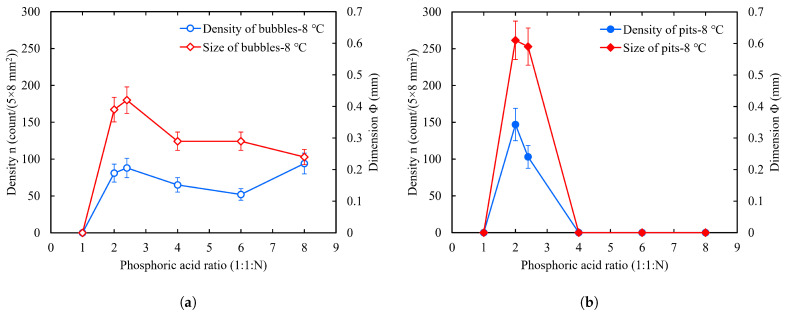
Effect of phosphoric acid ratio on the number and size of bubbles (**a**) and W-shaped pits (**b**) when the acid temperature was 8 °C and the rotor speed was 300 RPM. The critical bubble size, Φ, refers to the average diameter of most of the bubbles on the sample surface when they had grown large enough to detach from the surface. Similarly, the size of pits refers to the average diameter of most of the pits on the sample surface, which is also denoted by Φ. For the ratios 1:1:2 and 1:1:2.4, the critical bubble size was taken as the bubble size at 5 min because the bubble attachment time was more than 5 min.

To compare the effects of changes in the phosphoric acid ratio, we performed statistics on the bubbles and W-shaped pits on the sample face-4; the results are shown in Figure 8. The following can be observed: (1) When the phosphoric acid ratio N = 1, there were no bubbles or W-shaped pits on the sample surface during the polishing process. (2) With an increase in the phosphoric acid ratio N, the size of the bubbles showed a decreasing trend and peaked between N = 1 and N = 4. However, the density decreased and then increased. Our observations show that, at high phosphate ratios, the growth period of bubbles is short and starts at different times. Many bubbles had already left the surface when we counted them, which may have resulted in a large deviation of the statistical quantities from the true values. (3) The density and size of the W-shaped pits also decreased with an increase in the phosphoric acid ratio N and peaked between N = 1 and N = 4. However, the difference is that there were no recognizable W-shaped pits on the surface after N ≥ 4.

Forming noticeable W-shaped pits requires both a sufficiently long bubble attachment time and a high etching or polishing rate, i.e., a sufficiently large pit depth. When HF/${\mathrm{HNO}}_{3}$/$H_{3}{\mathrm{PO}}_{4}$ = 1:1:N, for a phosphoric acid ratio between N = 1 and N = 4, the bubble attachment time is long, the etching rate is high, and therefore it is very easy to form a deep pit. For N ≥ 4, a slow etching rate and a short attachment time make it difficult to form more obvious W-shaped pits on the sample surface. Experimental observations showed that the attachment time of bubble growth decreased with an increase in phosphoric acid ratio. For large cavities with lower frequencies, some labs use acid ratios of 1:1:4 to 1:1:8 with slower polishing rates to ensure polishing uniformity due to the long acid fill time [41]. However, if the etching rate is increased, such as by increasing the acid temperature, visible pits will still be formed at the bubble locations, so there is a risk of forming visible pits for N ≥ 4. It should be emphasized that the statistical pits refer to the W-shaped pits that are clearly recognizable when the samples are optically inspected.

The NO gas produced during the reaction of niobium with nitric acid has very low solubility in the mixed acids with high concentrations of viscous phosphoric acid (85%) [42,43]. In the ratio 1:1:N series, the NO gas production rate may always be greater than the dissolution rate. At high phosphoric acid ratios, the high acid viscosity makes gas diffusion and bubble movement difficult [44,45,46,47], which is favorable for gas aggregation on the niobium surface, the formation of bubbles, and their attachment to the surface. Conversely, at low phosphoric acid ratios, although the reaction is very fast, it is difficult for bubbles to form or attach to the surface. Moreover, the high viscosity inhibits disturbance and turbulence on the niobium surface [48,49], which makes bubbles more stably attached to the surface. Therefore, increasing phosphoric acid promotes bubble formation and attachment on the niobium surface.

In conclusion, reducing the phosphoric acid ratio to N ≤ 1 (with an acid ratio of HF (49%)/${\mathrm{HNO}}_{3}$ (69%)/$H_{3}{\mathrm{PO}}_{4}$ (85%) of 1:1:N) is favorable for BCP, resulting in no bubbles or W-shaped pits on the sample surface. When the acid temperature is 8 °C and the acid flow rate is 300 RPM, 1 < N < 4 is a dangerous range for the phosphoric acid ratio, which may lead to a large number of bubbles and W-shaped pits forming on the niobium surface. When N ≥ 4, a large number of bubbles form on the sample surface, even though there are no more obvious pits formed on the surface.

#### 2.4.2. Acid Temperature

Acid temperature plays an important role in chemical polishing. In the acid temperature experiments of sample BCP, the acid ratio was set to 1:1:2.4 due to this resulting in more noticeable bubbles and W-shaped pits; the rotor speed was 300 RPM, the polishing time was 5 min × 2, and the other experimental parameters were the same as before. The acid temperature was gradually increased from 6 °C to 22 °C with a temperature step of 2 °C, and finally to 25 °C. In BCP, 10∼25 °C is commonly the actual acid temperature of the cavity surface [50]. Three samples for each group of experiments (including one each of slightly rough fine-grain NF, large-grain NL, and fine-grain F) were simultaneously polished on the cleaning basket, and the polishing process was recorded with a camera.

**Figure 9:**
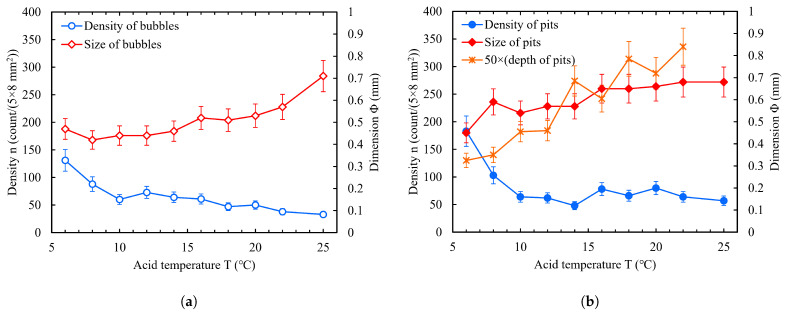
Effect of acid temperature on number and size of bubbles (**a**) and W-shaped pits (**b**) when acid ratio was 1:1:2.4 and rotor speed was 300 RPM.

The statistics for the bubbles on the sample top surface (face-4) for the first 5 min of BCP are shown in Figure 9a. With an increase in acid temperature, the density of critical bubbles on the sample surface decreased gradually; however, their size increased. After BCP, the surface of the samples was optically inspected using a high-definition camera in an ISO7 clean room. Figure 9b shows the statistical results for the density and size of the W-shaped pits on the face-4 for the experimental samples. On the whole, the number of pits showed a decreasing trend with increasing temperature. At acid temperatures of 10∼25 °C, there was no significant change in the pit density, while the size and depth of the pits increased with increasing temperature. The bubbles and pits showed the same trend with acid temperature.

**Figure 10:**
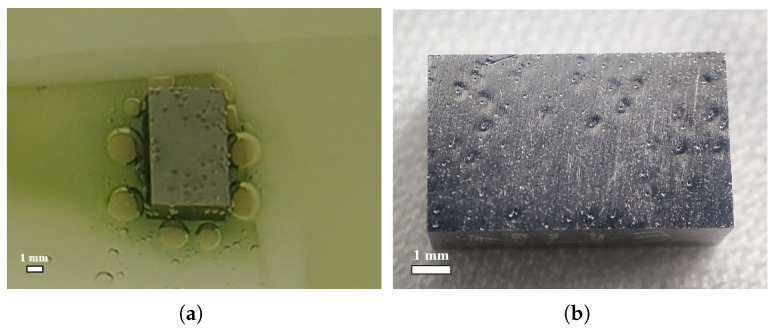
(**a**) Bubbles on face-4 after polishing for 270 s and (**b**) W-shaped pits on face-4 after polishing for large-grain sample NL134 when acid ratio was 1:1:2.4 and acid temperature was T = 20 °C.

Table 3 presents the polishing rates of large-grain samples at different temperatures. Figure 10 shows the bubbles and corresponding pits on the surface of the large-grain sample NL134, which was etched at an acid temperature of T = 20 °C. For NL134, the critical bubbles appeared to be larger and the W-shaped pits were larger and deeper compared to those for the previous large-grain sample NL133 that was etched at an acid temperature of T = 8 °C.

**Figure 11:**
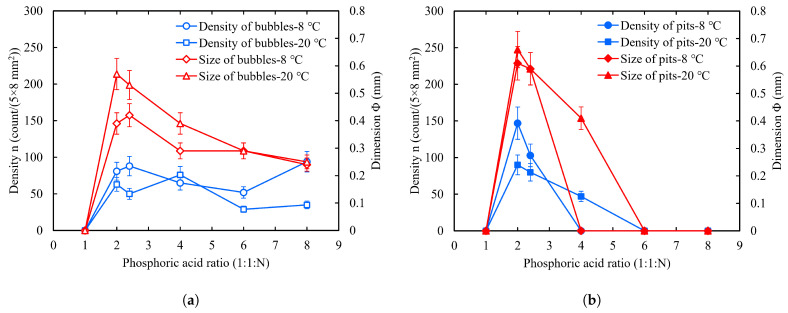
(**a**) Statistical results for bubbles and (**b**) W-shaped pits produced with different acid ratios at temperatures of 8 °C and 20 °C, respectively. Rotor speed was 300 RPM.

In addition to investigating the effect of acid temperature, we investigated the influence of acid ratios. Figure 11 shows the statistics for the bubbles and W-shaped pits on the sample face-4 produced with different acid ratios at temperatures of 8 °C, respectively. Consistent with the results for 1:1:2.4, the density of critical bubbles and pits decreased with temperature, while the size increased. In Figure 11b, we also observe that, for a phosphoric acid ratio of N = 4, pits reappeared on the surface after increasing the acid temperature from 8 °C to 20 °C. This suggests that increasing the acid temperature exacerbates etching at the bubble attachment site, i.e., promotes the formation of W-shaped pits. However, for a phosphoric acid ratio of N ≤ 1, no bubbles or pits appeared on the surface even when the acid temperature was increased to 20 °C.

Acid viscosity increases at lower temperatures, which is especially noticeable at high phosphoric acid ratios [51,52]. This hinders gas diffusion or bubble movement [44,45,46,47], and promotes gas aggregation on the niobium surface to form bubbles. In addition, the larger the bubble size, the higher the probability of neighboring bubbles merging. Therefore, the density of bubbles and W-shaped pits is large at low acid temperatures.

Increasing the acid temperature increases the chemical etching rate and gas yield (see Table 3), while the solubility of the produced NO gas in the acid decreases [42,53], leading to an increase in the bubble size due to more undissolved gas, and increases in the size and depth of the pits for the same attachment time. Experimental observations showed that, with a ratio of 1:1:2.4, the bubble attachment time on the experimental sample surfaces was more than 5 min at acid temperatures ranging from 6 °C to 25 °C, so the bubble attachment time on surface-4 was 5 min for all of them. Therefore, in BCP processes, the acid temperature should be lowered to suppress the formation of pits.

In summary, lowering the BCP acid temperature can suppress the formation of W-shaped pits, which is favorable for improving the Nb surface smoothness. Although a low acid temperature may increase the density of critical bubbles and W-shaped pits on the niobium surface, their size and depth decrease significantly.

#### 2.4.3. Acid Flow Velocity

BCP is an exothermic process that usually requires stirring acid or circulating acid to avoid high temperatures at the etching region. To investigate the effects of acid flow on bubble and W-shaped pit formation, we set the acid ratio to 1:1:2.4, acid temperature to 8 °C, and polishing time to 5 min × 2 and left the other experimental conditions unchanged. The experiments were divided into two groups: One involved a cleaning basket (i.e., the samples were placed on the cleaning basket for polishing, which is the standard method), and the rotor speed was set to 0 RPM, 100 RPM, 200 RPM, or 300 RPM, respectively. To obtain a greater variation in flow velocity, the cleaning basket was removed. Therefore, the second group of experiments were performed without a cleaning basket (i.e., the samples were placed on the beaker bottom for polishing), and the rotor speed was set to 100 RPM, 200 RPM, 400 RPM, or 600 RPM, respectively. In each group, three types of samples were polished at once, including slightly rough fine-grain NF, large-grain NL, and fine-grain F.

**Figure 12:**
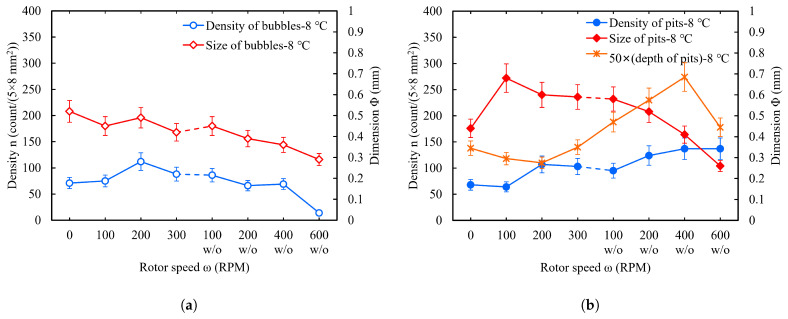
Effects of acid flow velocity on the number and size of bubbles (**a**) and W-shaped pits (**b**) when the acid ratio was 1:1:2.4 and the acid temperature was 8 °C.

Table 4 presents the polishing rates of large-grain samples at different rotor speeds and the corresponding acid flow velocities on the surfaces of the samples. Figure 12a shows the density and size statistics for critical bubbles on the sample surface (face-4) at different acid flow velocities. One can see that there was no significant variation in the density of critical bubbles within an acid flow velocity range of 0 RPM to 400 RPM (w/o), while the critical bubble size decreased with acid flow velocity. At acid flow velocities of 400 RPM (w/o) and 600 RPM (w/o), the faster acid flow caused many small bubbles to detach from the surface prematurely and hindered the identification of small bubbles, so the statistical density was smaller than the actual value. It should be noted that, even if the acid flow velocity exceeded 11.8 cm/s (≥400 w/o), there were still a large number of bubbles attached to the surface.

The statistics for the W-shaped pits on the sample surface (face-4) after BCP are shown in Figure 12b. As the acid flow velocity was increased from 0 RPM (stationary) to 600 RPM (w/o), the pit density increased while the pit size decreased. We also measured the depth of the W-shaped pits. As shown in Figure 12b, the pit depth increased with increasing flow velocity and became shallower after the velocity reached 600 RPM (w/o). With a flow velocity of 0 RPM, typical pits were not recognized and were replaced by anomalous pits at the sample edge, so the statistical pit size was smaller than the 100 RPM one.

**Figure 13:**
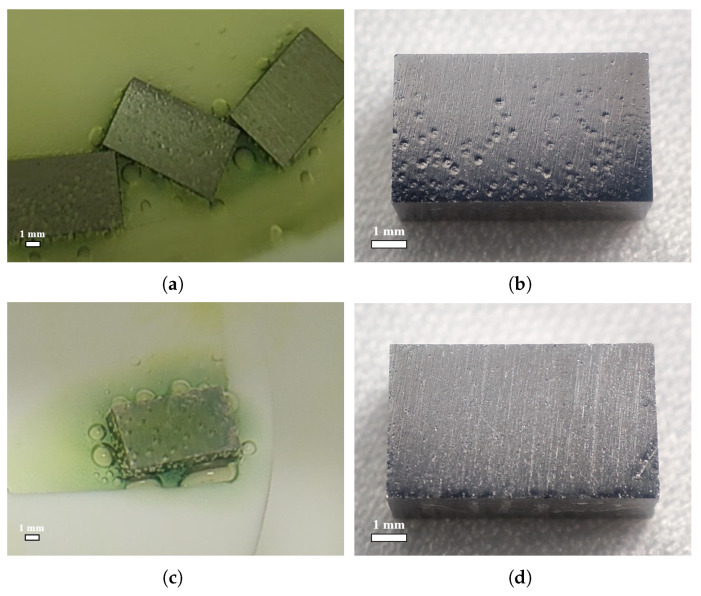
(**a**) Bubbles on face-4 after polishing for 270 s in the first 5 min of BCP and (**b**) W-shaped pits on face-4 after polishing for the large-grain sample NL148 (**middle**), when the rotor speed was 400 RPM (w/o). (**c**) Bubbles on face-4 after polishing for 270 s in the first 5 min of BCP and (**d**) W-shaped pits on face-4 after polishing for the large-grain sample NL143, without acid flow.

Figure 13a,b show the surface bubbles and W-shaped pits of the large-grain sample NL148 at an acid flow velocity of 400 RPM (w/o). Compared to those of the large-grain sample NL133 at an acid flow velocity of 300 RPM, sample NL148 had smaller critical bubble and pit sizes and a greater pit density and depth. A single-sided polishing rate of about 1.7 μm/min for sample NL148 made the typical pits more pronounced, with the deepest pit at around 13.7 μm.

Figure 13c,d show the bubbles and W-shaped pits on the sample surface without acid flow. Compared with those for the previous large-grain sample NL133, firstly, the bubbles on the surface of the large-grain sample NL143 grew much faster, and the attachment time to reach the critical bubble size was only ∼140 s. Secondly, the surface of NL143 was covered with a clearly green layer but the edges were relatively thin. Finally, the middle region of the polished surface had no visible W-shaped pits or imprints, and the deeper pits were mainly located at the edges.

This indicates that the green material accumulated on the surface can inhibit the etching of the bubble attachment location. As shown in Figure 13c,d, even though the bubbles in the area covered by the green solution had an attachment time of more than 270 s, there were no visible W-shaped pits formed at the bubble locations. In contrast, recognizable pits were formed at the sample edges with only a thin green solution layer. This green solution is similar to the viscous layer in the EP process, which restricts mass diffusive transport [54].

A larger acid flow velocity can carry away the produced gases faster and thus reduce bubble aggregation. In addition, the larger transverse thrust of the acid can make the bubbles detach from the surface at a smaller size [55,56]. Therefore, the size of the bubbles and pits decreased with acid flow velocity. There was a merging of neighboring bubbles during bubble growth, especially in the early stages when the density of small bubbles was large. The extent of bubble merging increased as the bubble size increased, so the bubble density on the sample surface decreased as the bubbles grew until the bubbles detached from the surface. Therefore, a large acid flow velocity corresponded to a high density of bubbles and pits.

A high acid flow velocity also led to an increase in the etching rate (see Table 4), so the pits became deeper as the flow velocity increased. However, when the flow velocity was high enough, the large transverse thrust on the bubbles resulted in very small bubbles with short attachment times, and the pits began to become shallower.

To sum up, increasing the acid flow velocity could reduce the size of the bubbles and W-shaped pits, which is favorable for BCP. Although the pit density increased with the flow velocity, a larger acid flow velocity could carry away the produced gases faster and thus reduce the bubble aggregation, and the larger transverse thrust of the acid could also make the bubbles detach from the surface at a smaller size.

#### 2.4.4. Grain Size

By comparing the density and size of bubbles and W-shaped pits on the three types of samples (NL, F, and NF), as well as the observation of the polishing process, we found that the density and size of bubbles and W-shaped pits formed on the surfaces of smooth large-grain samples (NL) and smooth fine-crystal samples (F) were basically the same.

**Figure 14:**
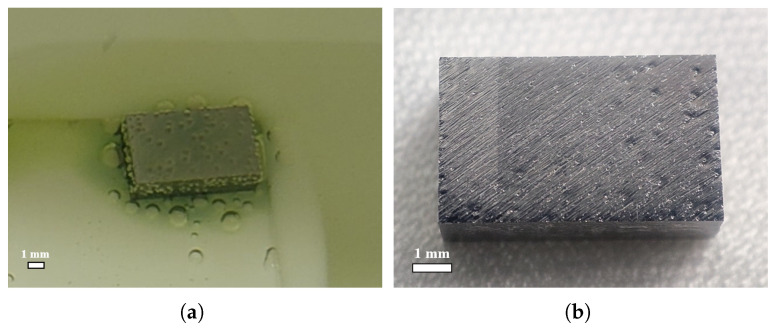
(**a**) Bubbles on face-4 after polishing for 270 s in the first 5 min of BCP and (**b**) W-shaped pits on face-4 after polishing for the large-grain sample NL135, when the acid ratio was 1:1:2.4, the acid temperature was 10 °C, and the rotor speed was 300 RPM.

In addition, the shape of the W-shaped pits gradually became blurred and irregular during polishing for fine-grain samples (see Figure 5), which made the identification of W-shaped pits more difficult. The grain boundaries seemed to have little effect on the distribution of bubbles and W-shaped pits. Figure 14 shows the bubbles and pits on the surface of the bicrystal sample NL135. There was no obvious tendency for bubbles and pits to cluster on the grain boundary or on both sides of the boundary.

For fine-grain materials, there is an etching difference or preferential etching in different grain orientations [13,39,57], causing the surface roughness to increase [2,58] and the W-shaped pits’ morphology to become irregular, which is a typical feature of BCP. For example, after the heavy polishing of fine-grain niobium samples, W-shaped pits formed in the initial stage are difficult to recognize and only a very rough surface is observed.

In conclusion, grain size does not significantly affect the formation of bubbles or W-shaped pits. Surface roughness and etching differences in different grain orientations increase the difficulty of identifying pits.

#### 2.4.5. Surface Orientation

The W-shaped pits’ distribution on the outer surface of a cavity after BCP-O is related to the surface orientation, and the inner surfaces of a cavity also have different orientations during BCP-I. Thus, it was necessary to investigate the effect of polished surface orientation on W-shaped pits. We selected one strip sample and suspended it individually on a cleaning basket for the sample BCP experiment to imitate the formation of bubbles and pits on the differently oriented surfaces during cavity BCP. To ensure that the conditions were consistent with those of the cavity BCP-O, the strip sample was polished under the following conditions: an acid ratio of 1:1:2, acid temperature of 10 °C, rotor speed of 100 RPM, and polishing time of 10 min. It is noted that, although the acid temperature for cavity BCP-O is usually set at 8 °C, the actual acid temperature in the polishing bath can reach 10∼13 °C. In addition, the polishing time was increased to 10 min to facilitate the identification of W-shaped pits.

**Figure 15:**
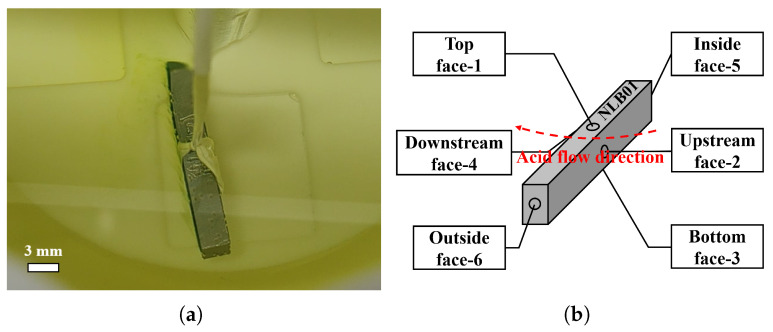
(**a**) Bubbles on the surface of the large-grain strip sample NLB01 suspended on a cleaning basket during BCP (t = 270 s), when the acid ratio was 1:1:2, the acid temperature was 10 °C, and the rotor speed was 100 RPM. (**b**) The six faces are defined as shown in the schematic.

Figure 15 presents the large-grain strip sample NLB01 under polishing, with the six faces representing the different orientation surfaces of the cavity. During polishing, it was found that the acid flow was very slow on the downstream face-4 and the inside face-5, accompanied by a surrounding green color solution. Initially, the density and size of the small bubbles on the inside and outside faces were comparable, but as the polishing continued, the inside face bubbles reached a critical size earlier and detached from the surface.

**Figure 16:**
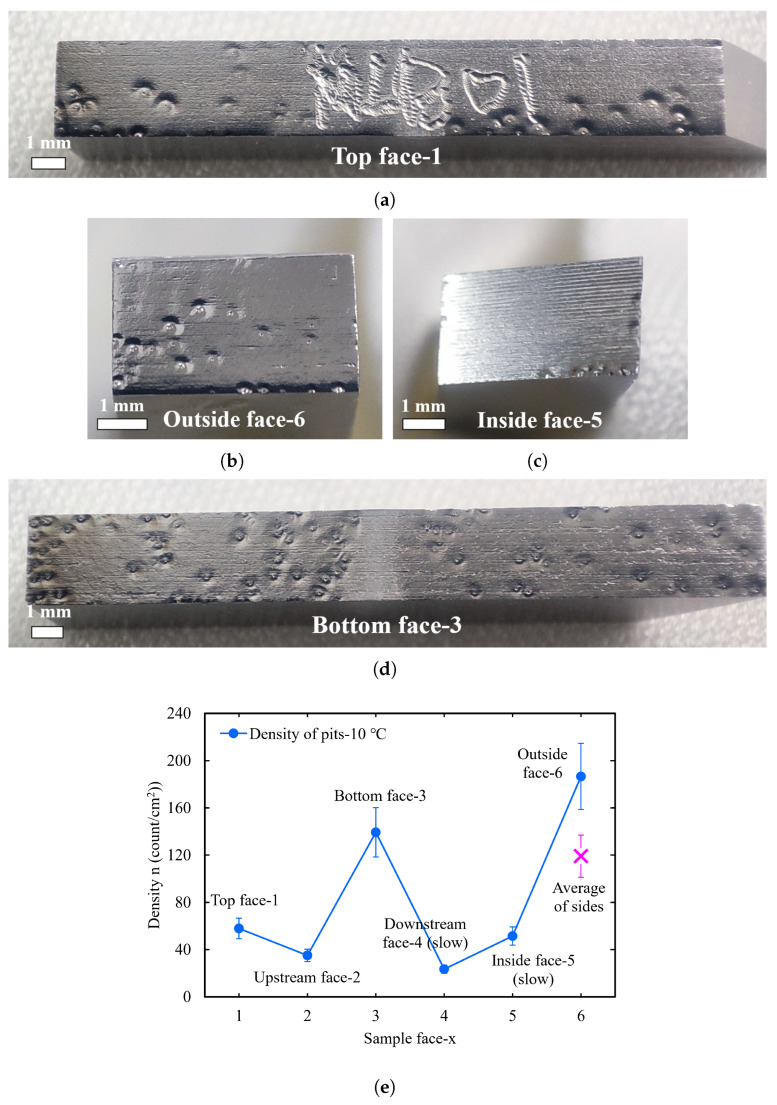
(**a**–**d**) Morphology of the top, side, and bottom faces of the large-grain strip sample NLB01. (**e**) W-shaped pit density versus polished surface orientation. For comparison with the top and bottom faces, “×” is the average pit density for the inside and outside faces.

Each face of the polished strip sample showed significant W-shaped pits, as shown in Figure 16. There were significant differences in the density of the pits on the six faces. The bottom face had very dense pits, while the downstream and inside faces were very sparse. Figure 16e presents the density of the W-shaped pits on the six faces of the large-grain strip sample NLB01, among which the top face had the smallest pit density, followed by the side face, and the bottom face had the largest pit density. This is consistent with the distribution trend of the W-shaped pits on the equator of the cavities after BCP-O.

According to the observations, some bubbles on the top face detached from the sample surface earlier during the bubble growth process. However, it may have been difficult for the bubbles on the bottom face to detach from the surface because they were blocked by the bottom face; thus, the bottom face had a large density of bubbles and pits. In addition, the W-shaped pit density differences on different faces were also affected by the acid flow velocity. There was a lower pit density on the inside face, the downstream face, and the area near the inside where the acid flow velocity was slower. This further confirms the role of acid flow velocity.

## 3. Mechanism of Bubble and Pit Formation

**Figure 17:**
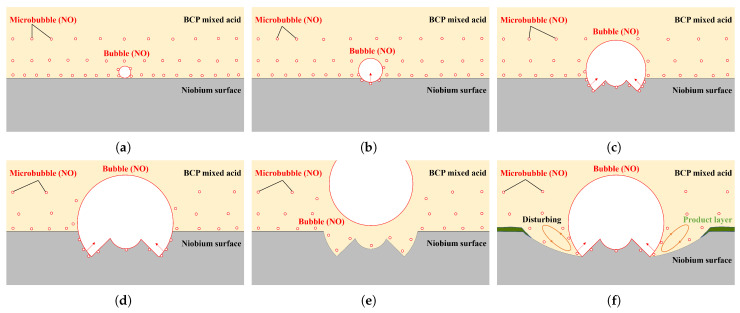
The proposed mechanism by which bubbles and W-shaped pits are formed: (**a**) A bubble nucleation center is formed on the niobium surface. (**b**) The bubble grows and etches a small U-shaped pit during the point etching stage. (**c**) The bubble continually grows and etches a W-shaped pit during the annular etching stage, where the slower acid exchange results in a slow etch rate in the inner region of the bubble, thereby forming an annular etch. (**d**) The bubble and pit continue to grow. (**e**) After reaching a critical size or being affected by other factors, the bubble detaches from the surface and leaves a typical W-shaped pit. (**f**) There is a larger annular pit due to gas perturbation.

Based on the bubble evolution process on the sample surface during BCP and the shape of the W-shaped pits on the polished surface, we propose a mechanism by which the bubbles and W-shaped pits are formed. Like the electrolytic gas evolution process, the formation of bubbles on the niobium surface also consists of three phases: nucleation, growth, and detachment [59,60,61]. In the initial stage of niobium’s reaction with acid, if the solubility of the produced NO gas in the acid is so low that the gas generation rate is greater than the dissolution rate (i.e., the produced gas is locally supersaturated on the niobium surface), then undissolved gas aggregates on the niobium surface and forms a bubble nucleation center, as shown in Figure 17a. The bubbles then grow by absorbing gases or merging with other bubbles as shown in Figure 17b–d. When the bubbles grow to the critical size and the pulling force is greater than the adhesive force, the bubbles detach from the niobium surface and enter the acid solution, as shown in Figure 17e.

Usually, homogeneous nucleation within the liquid bulk and even heterogeneous nucleation on molecular smooth surfaces require very high levels of gas supersaturation. However, at low levels of supersaturation, bubbles can also nucleate on vessel surfaces in contact with liquids, at pre-existing sub-stable gas caves, on suspended particles, or from sub-stable microbubbles in the liquid bulk [60]. The formed bubbles on the niobium surface during the BCP process are most likely a result of heterogeneous nucleation due to localized supersaturation of produced NO gas on the niobium surface. In addition, the gas is carried away slowly by the acid at a small acid flow velocity, which makes gas supersaturation easier. For example, the slow acid exchange at the sample bottom tends to form bubbles, which merge and form big bubbles around the sample, discharged from the bottom (see Figure 7a).

Bubble formation and attachment on the niobium surface may also be related to acid viscosity and disturbance. A high acid viscosity hinders gas diffusion or bubble movement [44,45,46,47], which favors the formation of bubbles through gas aggregation on the niobium surface and the attachment of bubbles to the surface. Niobium surface disturbance and turbulence are also suppressed by a high acid viscosity [48,62,63], which causes bubbles to attach more stably to the surface.

At the three-phase interface between the bubble, acid, and niobium, the chemical etching rate of niobium is probably faster than that of the surrounding area, and the difference in etching rate causes the gradual formation of a pit at this location [35,39]. This is likely due to a mass transport channel without a salt film being formed at the bubble location, which promotes ${{\mathrm{NO}}_{3}}^{-}$ and $F^{-}$ diffusive transport and reaction with the Nb substrate [54,64]. A salt film such as the green product layer limits ion transport diffusion, thereby inhibiting the reaction rate [54]. The gas generated from the reaction of a large number of ${{\mathrm{NO}}_{3}}^{-}$ ions with Nb causes strong perturbation or agitation, which further accelerates the transfer of reactants and products there [65,66].

The formation process of the W-shaped pit is shown in Figure 17. In the first stage of bubble growth, the three-phase interface can be approximated as a point at which a small etching pit with a U-shaped cross-section is formed, and thus, we refer to this stage as the point etching stage (see Figure 17b). As the bubble grows, the interface gradually expands from a point to a circle. The slower acid exchange results in a slow etch rate in the inner region of the circle, while the etching rate on the circle remains fast, thus forming an annular etching pit (Figure 17c), and the bubble growth enters the second stage. This stage forms a ring-shaped pit with a growing size and a W-shaped cross-section, which we call the annular etching stage (Figure 17c,d). When the bubble size reaches a critical maximum or the stable attachment conditions are disrupted by other factors, the bubble detaches from the niobium surface and enters into the acid [61], and a typical W-shaped pit is left on the niobium surface at the location of the bubble’s attachment (Figure 17e).

In fact, in addition to affecting the etching rate at the attachment location, bubbles can also significantly affect the surrounding area. A faster etching rate at the bubble attachment site results in the formation of a strong perturbation or stirring in the neighboring area around the bubble (see Figure 17f). Stirring may increase the reaction rate by increasing the transfer rate for reactive substances and preventing the accumulation of reaction products on the niobium surface [67]. Closer to the bubble, the perturbation is stronger and the etching rate is higher, so the pit size is usually larger than the bubble–niobium contact interface size. A relatively typical and visual example of bubbles affecting neighboring regions is the presence of transparent annular regions around some bubbles as shown in Figure 6 and Figure 14, which is particularly evident when the polishing rate is moderate, such as for 1:1:2 or 1:1:2.4, and the acid flow rate is slightly slower.

Based on the previous experimental results and the proposed mechanism, we propose two methods for preventing the W-shaped pit formation: (1) removing bubbles, such as by preventing the bubbles’ formation or directly removing the bubbles on the niobium surface through stirring, vibrating, or rotating during polishing; (2) increasing the NO gas dissolution rate in the acid and reducing the production rate, while reducing the acid viscosity, via changing the acid ratio or lowering the acid temperature, etc. From this perspective, we can optimize the BCP process by reducing the acid temperature (e.g., 8 °C), appropriately increasing the acid flow velocity (e.g., 1.3 cm/s), or even changing the acid ratio of BCP (e.g., reducing the phosphoric acid ratio to N ≤ 1). In terms of BCP device improvement, we can add a mechanical stirring or vibration device, or a rotating device similar to the horizontal EP, to remove the bubbles on the niobium surface during polishing.

## 4. Discussion

During the polishing process, few new bubbles were formed in the subsequent stages, including the second 5 min of BCP, compared to the initial stage. This phenomenon is likely due to the removal of the highly reactive superficial layer of the initial few microns on the surface, which has a large etching rate [8,9,39]. Along with the removal of the superficial layer and increase in the niobium concentration in the acid solution, the polishing rate decreased in the subsequent stages, which caused the gas saturation to decrease and made the formation of bubbles more difficult. This is consistent with experimental observations. With an acid temperature of 8 °C, rotor speed of 300 RPM, and ratio of 1:1:2, new bubbles basically ceased to form on the sample surface after polishing for about 1 min, i.e., when a ∼1.2 μm thin layer had been removed. At a ratio of 1:1:8, new bubbles still appeared on the sample surface even after polishing for 4 min, i.e., when a ∼0.7 μm thin layer had been removed.

In addition, our experiments show that the surface roughness and damage layer seemed to have no significant effect on the formation of bubbles. For example, many W-shaped pits still formed on the surface of the large-grain cavity L03 after BCP treatment with an acid ratio of 1:1:2.4, which had previously been treated with a bulk EP of 170 μm (see Figure 1b). The surface roughness only affects the recognition of W-shaped pits, while the damage layer with a high etching rate may exacerbate the bubble and pit formation.

The effects of the phosphoric acid ratio, acid temperature, and acid flow velocity on pit formation might be explained by a diffusion-limited mechanism. During BCP, the etching or dissolution of niobium may be both diffusion-limited and chemically controlled [11,39,48]. When the ratio of HF and ${\mathrm{HNO}}_{3}$ is fixed and assuming that the dissolution process is diffusion-limited, the niobium dissolution rate can be determined using Fick’s law [39].

(1) $$\frac{dn}{dt}=DA\frac{c}{\delta }$$

where *A* is the surface area of the sample, *D* is the diffusion coefficient of the reactants in the acid, *c* is the concentration of the reactants in the acid, and δ is the thickness of the reactant depletion layer near the niobium surface. Upon lowering the acid temperature and increasing the acid viscosity by adding phosphoric acid, the diffusion coefficient *D* of the reactants reduces and, thus, the dissolution rate decreases [39,68]. Increasing the acid flow velocity and stirring can reduce the thickness δ of the depletion layer, which increases the dissolution rate [39,68]. The W-shaped pit formation may also be related to the depletion layer being disrupted or thinned due to increased stirring at the bubble location [36], whereas the surface outside the pit is covered by the depletion layer and therefore dissolves slowly [35].

The classical ratio of 1:1:2 is widely used globally; however, there are not many reports of W-shaped pits on the cavity surface after BCP treatment. This may due to the following: (1) The concentration of hydrofluoric acid and nitric acid used is lower. For example, the American laboratories JLAB, FNAL, ANL, etc., use hydrofluoric acid at 49% and nitric acid at 69% [11,69,70,71], but the European laboratories DESY, CEA, and INFN use hydrofluoric acid at 40% and nitric acid at 65% [12,21,24,65]. (2) Methods such as agitation, rotation, or forced convection have been used to remove surface bubbles or prevent bubble formation [11,67,72]. (3) The polishing process parameters differ. For example, injected acid temperatures as low as 5 °C [72,73,74] and low acid flow velocities have been used [69,72,74]. (4) A high phosphoric acid ratio may be used. Some labs use 1:1:4 to 1:1:8 for polishing uniformity [6,75,76]. Large cavities at low frequencies need long acid fill times, and the bottom of the cavity is etched more than the top, so slower etch rates are effective in alleviating the problem [41]. (5) The effect of the W-shaped pits on the cavity performance at low and medium acceleration gradients is not significant and, therefore, is not discussed in some research [24,25].

Based on our studies, the BCP treatment of cavities can be optimized in the following ways. Reducing the phosphoric acid ratio to N ≤ 1 is critical to avoid bubble formation. Hollow V-shaped seats can be used to support the cavity for BCP-O, and the various couplers of the cavity can be connected to the acid circulation branches for BCP-I to promote local acid flow, thereby preventing local over-temperature and reducing the local gas saturation level. In addition, properly increasing the polishing flow rate, lowering the initial acid temperature, and water-cooling the outer wall of the cavity are helpful for mitigating the pitting caused by bubbles.

Bubbles and W-shaped pits show one-to-one correspondence, and therefore, ideally, the two statistics should be consistent. However, the actual bubble statistics can only reflect the surface situation of the sample at a certain moment in the polishing process. The pit statistics are the total W-shaped pits formed throughout the reaction process and can only reflect the bubbles that can form obvious pits on the sample surface. Therefore, this incomplete reflection makes the density statistics of critical bubbles and W-shaped pits somewhat different.

In the acid flow velocity experiments, the circular flow of acid in the beaker causes vortices at high rotor speeds, which may limit the range of flow velocities studied. In addition, using or not using cleaning baskets resulted in the two classes of experiments not being exactly the same, affecting the statistical results.

Next, we will carry out experiments related to changing the acid ratio and removing bubbles through vibration to further verify our mechanism and solutions, and try to essentially solve the problem of W-shaped pits.

## 5. Conclusions

We investigated the formation of special W-shaped pits on the niobium surface during the BCP process through sample BCP experiments and surface optical inspection. We found that the direct cause of W-shaped pit formation on the surface of niobium samples or niobium cavities after BCP is the formation and attachment of bubbles on the niobium surface during the polishing process.

We found that the formation of bubbles and W-shaped pits is affected by multiple factors, including the phosphoric acid ratio, acid temperature, acid flow velocity, and polished surface orientation, but is independent of grain size. Reducing the phosphoric acid volume ratio to N ≤ 1 (with a volume ratio of HF (49%)/${\mathrm{HNO}}_{3}$ (69%)/$H_{3}{\mathrm{PO}}_{4}$ (85%) = 1:1:N) is favorable for BCP. For the phosphoric acid ratio, a range of 1 < N < 4 could be dangerous, leading to a large number of bubbles and W-shaped pits forming on the niobium surface. Lowering the acid temperature can suppress the formation of W-shaped pits and significantly reduce the size and depth of the pits, which is beneficial for improving the BCP surface quality. Increasing the acid flow velocity can reduce the size of bubbles and W-shaped pits. Having the polished surface facing down promotes bubble aggregation and increases the W-shaped pit density.

Finally, we propose a mechanism by which the bubbles and W-shaped pits are formed on the niobium surface, in which the bubble formation process can be divided into nucleation, growth, and detachment. The bubble formation may be caused by the local supersaturation of the produced gas on the niobium surface. Bubble formation and attachment are affected by acid viscosity and disturbance. The possible mass transport channel formed at the bubble location accelerates the etching, which leads to the formation of W-shaped pits.

## Figures and Tables

**Table 1:** Number and dimension (X direction) of pits on face-4 of samples NL131, F131, and NF131.

Samples	Number of Pits	Diameter of “W” Pit, Φ (mm)	Depth of “W” Pit, H (μm)	Diameter of Bump, d (mm)	Diameter of Small Point Pit, ϕ (mm)	Depth of Small Point Pit, h (μm)	Cross-Sectional Shape of Pits
NL131	147 ± 8	0.61 ± 0.03	12.1 ± 0.1	0.17 ± 0.01	0.06 ± 0.01	5.5 ± 0.1	W
F131	141 ± 9	0.67 ± 0.04	9.9 ± 0.2	0.23 ± 0.02	0.06 ± 0.01	6.8 ± 0.2	W
NF131	135 ± 17	0.69 ± 0.04	10.4 ± 0.2	0.21 ± 0.02	0.08 ± 0.01	6.3 ± 0.2	W

**Table 2:** Effect of phosphoric acid ratio on polishing rate.

Acid Ratio HF/${\mathrm{HNO}}_{3}$/$H_{3}{\mathrm{PO}}_{4}$	Rotor Speed (RPM)	Acid Temperature ^1^ (°C)	Polishing Rate (μm/min)
1:1:1	300	8	2.79 ± 0.05
1:1:2	300	8	1.21 ± 0.04
1:1:2.4	300	8	1.05 ± 0.07
1:1:4	300	8	0.44 ± 0.08
1:1:6	300	8	0.23 ± 0.07
1:1:8	300	8	0.17 ± 0.04

^1^ Acid temperature fluctuation: ≤0.5 °C.

**Table 3:** Effect of acid temperature on polishing rate.

Acid Temperature ^1^ (°C)	Rotor Speed (RPM)	Acid Ratio HF/${\mathrm{HNO}}_{3}$/$H_{3}{\mathrm{PO}}_{4}$	Polishing Rate (μm/min)
6	300	1:1:2.4	0.90 ± 0.04
8	300	1:1:2.4	1.05 ± 0.07
10	300	1:1:2.4	1.08 ± 0.10
12	300	1:1:2.4	1.11 ± 0.05
14	300	1:1:2.4	1.19 ± 0.14
16	300	1:1:2.4	1.25 ± 0.10
18	300	1:1:2.4	1.28 ± 0.12
20	300	1:1:2.4	1.29 ± 0.13
22	300	1:1:2.4	1.33 ± 0.05
25	300	1:1:2.4	1.46 ± 0.04

^1^ Acid temperature fluctuation: ≤0.5 °C.

**Table 4:** Effect of acid flow velocity on polishing rate.

Rotor Speed (RPM)	Acid Flow Velocity ^2^ (cm/s)	Acid Temperature ^3^ (°C)	Acid Ratio HF/${\mathrm{HNO}}_{3}$/$H_{3}{\mathrm{PO}}_{4}$	Polishing Rate (μm/min)
0	∼0	8	1:1:2.4	0.95 ± 0.04
100	∼0.6	8	1:1:2.4	0.95 ± 0.08
200	∼0.9	8	1:1:2.4	1.06 ± 0.13
300	∼1.3	8	1:1:2.4	1.05 ± 0.07
100 w/o ^1^	∼1.7	8	1:1:2.4	1.20 ± 0.22
200 w/o	∼4.4	8	1:1:2.4	1.42 ± 0.07
400 w/o	∼11.8	8	1:1:2.4	1.72 ± 0.12
600 w/o	∼17.7	8	1:1:2.4	1.74 ± 0.12

^1^ w/o: without a cleaning basket. ^2^ Acid flow velocity: the velocity on the sample surface. ^3^ Acid temperature fluctuation: ≤0.5 °C.

## Data Availability

The original contributions presented in this study are included in the article. Further inquiries can be directed to the corresponding author.

## Abbreviations

The following abbreviations are used in this manuscript:

BCP	Buffered chemical polishing
BCP-O	Outer-surface BCP
BCP-I	Inner-surface BCP
SRF	Superconducting radio-frequency
RRR	Residual resistivity ratio
EP	Electropolishing
RPM	Revolutions per minute
